# Pretreatment Intra-Voxel Incoherent Motion Diffusion-Weighted Imaging (IVIM-DWI) in Predicting Induction Chemotherapy Response in Locally Advanced Hypopharyngeal Carcinoma

**DOI:** 10.1097/MD.0000000000003039

**Published:** 2016-03-11

**Authors:** Wei Guo, Dehong Luo, Meng Lin, Bing Wu, Lin Li, Yanfeng Zhao, Liang Yang, Chunwu Zhou

**Affiliations:** From the Department of Diagnostic Radiology, Peking Union Medical College, Cancer Institute & Hospital, Chinese Academy of Medical Sciences, Beijing, P.R. China.

## Abstract

The aim of this study was to predict response to induction chemotherapy in patients with locally advanced hypopharyngeal carcinoma by IVIM values.

Twenty-eight patients with locally advanced hypopharyngeal carcinoma underwent IVIM studies using 12 different *b* values (*b* = 0, 10, 20, 30, 50, 70 100, 150, 200, 400, 800, and 1000 s/ mm^2^). All patients underwent 2 MRI studies: a baseline exam before any treatment and a mid-treatment exam 3 weeks after induction chemotherapy. In the IVIM approach, *D*^∗^, *f*, and *D* were extracted from a bi-exponential fit. For comparison, the ADC map were extracted from a mono-exponential fit. At the end of induction chemotherapy, patients were classified as responders or nonresponders group according to the Response Evaluation Criteria in Solid Tumors criteria (RECIST), based on their MRI measurement. The patients were classified into high grade group (G1), moderate grade group (G2), and low grade group (G3) according to the tumor pathological grading. The predictive value of IVIM parameters were examined with Student's *t* test, analysis of variance (ANOVA), and receiver operating characteristic (ROC) curves.

After 2 cycles of induction chemotherapy, 18 patients were categorized into the responder group whereas the other 10 patients were considered nonresponders. Compared with the pretreatment value, the post-treatment ADC value and *D* value was significantly higher and the posttreatment *D*^∗^ value was significantly lower (all *P* <  0.05). In contrast, post-treatment *f* parameter only changed slightly (*P* > 0.05). Compared with nonresponders, a notably lower pretreatment ADC value, *D* value, posttreatment *D*^∗^ value, and higher posttreatment ADC value, *D* value, ΔADC, Δ*D*, and Δ*D*^∗^ were observed in responders (all *P* < 0.05), but no significant change in Δ *f* among the 2 group (*P* > 0.05). The ROC curve analysis indicated that the cutoff of pretreatment *D* value in best predicting tumor's chemotherapeutic response was 0.847 × 10^−3^ mm^2^/s, and the corresponding AUC, sensitivity, and specificity were 0.806, 75.0%, and 88.9%, respectively. Although pretreatment IVIM-derived parameters had no significant differences between high grade, moderate grade, and low grade group, a trend towards lower *D*^∗^ was observed with increasing tumor grading from G3 to G1.

IVIM-DWI can potentially predict the treatment response to induction chemotherapy for hypopharyngeal carcinoma.

## INTRODUCTION

Hypopharyngeal carcinoma constitutes ∼3% to 5% of all malignancies in the head and neck squamous cell carcinoma (HNSCC).^1^ Nearly 80% of hypopharyngeal tumors arise from pyriform sinus, which is the most common subsite, 20% arise from the posterior pharyngeal wall and the post-cricoid region.^2^ Most patients of hypopharyngeal carcinoma present with significant comorbidities and advanced-stage disease. The overall survival is relatively poor because of high rates of regional and distant metastasis. Induction chemotherapy results in a decline in risk of distant metastasis and an upsurge in organ preservation in patients with hypopharyngeal carcinoma. However, methods for standardizing and assessing treatment response of chemotherapy regimen, especially induction chemotherapy, remain controversial.^3,4^ Induction chemotherapy before concomitant CRT cannot be routinely recommended for patients with locally advanced hypopharyngeal carcinoma. It may serve as a prognostic tool with potential to alter subsequent therapy based on response. Resistance to chemoradiation is widely recognized as the main cause of relapse for locally advanced hypopharyngeal carcinoma. Thus, it is important to develop prognostic imaging biomarkers that can accurately predict treatment outcome before initiation of treatment. These imaging biomarkers may help in stratifying patients who would benefit from chemoradiation therapy from those who would not. For nonresponders, alternative treatment strategies such as upfront neck surgery and other novel treatment modalities that include biotherapy and immunotherapy can be recommended for patients.^5^ So, it is meaningful to assess the treatment response of induction chemotherapy to optimize the treatment regimen individually.

Advanced imaging techniques are increasingly applied in HNSSC. Merging evidence suggests that diffusion-weighted magnetic resonance imaging (DWI-MRI) improves tissue characterization, staging, and response to treatment in HNSSC.^6–10^ Conventional diffusion-weighted imaging (DWI), with a mono-exponential model, could not separate perfusion and true diffusion-related effect. This could be resolved in intra-voxel incoherent motion (IVIM) with a bi-exponential model.^11^ The IVIM imaging is characterized by 3 parameters: pure diffusion coefficient (*D*); microvascular volume fraction (*f*); and perfusion-related incoherent microcirculation (*D*^∗^).^12^ IVIM-derived parameters may characterize the actual status of diffusion in tumors more accurately than conventional DWI because it provides both perfusion and true diffusion-related measurements and its effectiveness has been explored in various tumor types, including head and neck tumors.^13–21^ IVIM-MRI has to date, rarely been used as a predictive tool to assess hypopharyngeal carcinoma treatment response to induction chemotherapy.

The present study was therefore designed to evaluate the potential of IVIM parameters (*f*, *D*, and *D*^∗^) in predicting the local treatment response of induction chemotherapy in locally advanced hypopharyngeal carcinoma.

## MATERIALS AND METHODS

### Patients and Treatment

This retrospective study was approved by the ethics committee of Cancer Hospital of Chinese Academy of Medical Sciences and written informed consent was obtained for patients enrolled in the study. Of the 32 patients who met the inclusion criteria (from December 2014 to October 2015), 28 patients (All for men, no women; age range, 44–72 years; mean age, 54.8 ± 18.4 years) eligible for this study met the following criteria: with a confirmed diagnosis of N2M0 or N3M0 squamous cell carcinoma(SCC) prior to MRI scan; clinically staged as the stage III or IV according to the 7th edition of American Joint Committee on Cancer Classification(AJCC);^22^ without any MRI contraindications (including claustrophobia, and metal foreign body, especially cardiac pacemaker); not allergic to gadolinium-based contrast agent; have received 2 whole cycles of induction chemotherapy before next treatment; and be willing to complete the follow-up IVIM-MRI scans. Of the other 4 patients were excluded from this study for the following reasons: severe image distortions (n = 1), and the length-diameter of primary leisons < 1.0 cm (n = 3). The primary tumor locations at initial presentation were pyriform sinus (22/28, 78.6%), posterior pharyngeal wall (4/28, 14.3%), and the post-cricoid region (2/28, 7.1 %). Distributions of pathological grade were: G1 (high grade, n = 8); G2 (moderate grade, n = 10); G3 (low grade, n = 10).

All patients received 2 cycles of induction chemotherapy. Induction chemotherapy regimen included paclitaxel (270 mg/m^2^ of body–surface area, for day 1), followed by intravenous cisplatin (40 mg/m^2^, for day 1–2). Induction chemotherapy was given every 3 weeks for 2 cycles. Response was evaluated ∼3 weeks after the second cycle of induction chemotherapy in all patients. Patients were classified as responders {CR (complete response) and PR (partial response)}, or nonresponders {(SD (stable disease) and PD (progression disease)} according to the RECIST Criteria.^23^

## IMAGING ACQUISITION

### Conventional MRI

All MRI imaging was conducted on a 3.0-T magnetic resonance system (GE Healthcare, Discovery 750) equipped with an 8-channel neurovascular phased-array coil. Before any treatments, all enrolled subjects received the following conventional MRI sequences: axial T_1_-weighted imaging with fast spin echo (Ax T_1_WI-FSE): repetition time/echo time (TR/TE)  =  660/9.3 ms, slice number  = 30, field of view (FOV)  =  230  ×  230 mm^2^, slice thickness/gap  = 5/1 mm; axial T2-weighted imaging with fast spin echo (Ax T_2_WI-FSE): TR/TE  = 5760/88.3ms, slice number = 30, FOV =  230 × 230 mm^2^, slice thickness/gap = 5/1 mm; and post-contrast enhanced acquisition of axial T_1_WI with fast spoiled gradient recalled echo (Ax FSPGR): TR/TE  =  7/2.9 ms, slice number  = 30, FOV =  230 × 230 mm^2^, slice thickness/gap  = 5/1 mm. For the postcontrast acquisition, Gadodiamide injection (Omniscan, GE Healthcare Ireland) was intravenously injected at a dose of 0.1 mmol/kg of body weight and a rate of 2.0 mL/s, followed by a 20-mL saline flush. The total scan time was ∼15 minutes.

## IVIM-MRI

All patients underwent 2 MRI studies, first study was prior to any treatment at the baseline and second study was performed within 3 weeks after completion of 2 cycle (42 days) induction chemotherapy. Acquisition of IVIM was performed using single-shot echo-planar imaging (EPI) with 3 orthogonal motion probing gradients. Twelve *b*-values (*b* = 0,10,20,30,50,70,100,150,200,400,800 and1000 s/mm^2^) were used. The corresponding parameters were as follows: TR/TE 2500/79 ms; Matrix 256  × 256; FOV 230 × 230 mm^2^; slice number = 30, slice thickness/gap = 5/1 mm; NEX is 2 for *b* value from 0 to 200 s/mm^2^ and 3 for *b* value above the range. Image acquisition was performed in the axial plane, covering the hypopharynx from the oropharynx to the clavicle region. The scan time was 5 minutes 8 seconds. Patients were instructed not to make any other voluntary motion and the ASSET (array spatial sensitivity encoding technique) was turned off before the IVIM scanning.

## IMAGE AND DATA ANALYSIS

For calculating the standard ADC value, a mono-exponential fitting of the data was calculated by a linear fit equation (S/ S_0_ = exp (−*b*^∗^ADC)) using all *b* values,^24^ where S_0_ is the signal without diffusion gradient and S is the signal with diffusion weighting.

All IVIM parameters were calculated from the signal intensity of multiple *b*-values, using the Levenberg–Marquardt nonlinear least squares algorithm.^25^ The bi-exponential model from an IVIM sequence was expressed by the following equation, as described by Le Bihan et al^11^: 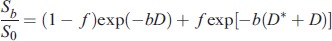


where *S*_*b*_ is the signal intensity at the *b* value of 0 to 1000, and *S*_0_ is the signal intensity at the *b* value of 0, *D* is the true diffusion coefficient, *f* is the fractional perfusion related to microcirculation, and *D*^∗^ is the pseudo-diffusion coefficient. *D* value was calculated by a simplified linear fit equation (*S*_*b*_ = *S*_0_ × exp^−*bD*^) using *b* values > 200 s/mm^2^. This was based on the assumption that *D*^∗^ is significantly greater than *D* such that its influence on signal decay can be neglected for *b* values > 200 s/mm^2^. The *f* and *D*^∗^ values were calculated by using a nonlinear regression algorithm for all *b* values.

IVIM-derived parameters were calculated and measured blindly by 2 independent radiologists with 5-year experience in head and neck imaging. ROIs for IVIM fitting were prescribed on primary tumors on DWI images (*b* = 800 s/mm^2^), avoiding visually large cystic or necrotic areas. The ROI was transferred automatically onto the ADC, *D*, *f*, and *D*^∗^ parametric maps by using MADC software (GE Healthcare, AW4.6 WorkStation), and the corresponding parameters in each ROI and also the average over all 3 ROIs were used for analysis, and each ROI area was 30 to 50 mm^2^. We used anatomical axial T_2_W images and enhanced T_1_W images as references to determine tumor areas on the corresponding DWI images.

## STATISTICAL ANALYSIS

All statistical analyses were performed using SPSS (version19; IBM SPSS; Chicago, IL), with a 2-tailed probability value, a *P* <  0.05 was considered statistically significant. The Kolmogorov–Smirnov test was done for each IVIM-derived parameter. The statistical method of unpaired *t* test, paired *t* test, and analysis of variance (ANOVA) were done for pretreatment and post-treatment IVIM-derived parameters. The receiver-operating characteristic (ROC) curve analysis was done for estimating predictive capability of IVIM-derived parameters.

## RESULTS

### Pretreatment IVIM-Derived Parameters in Different Response Groups

**FIGURE 1:**
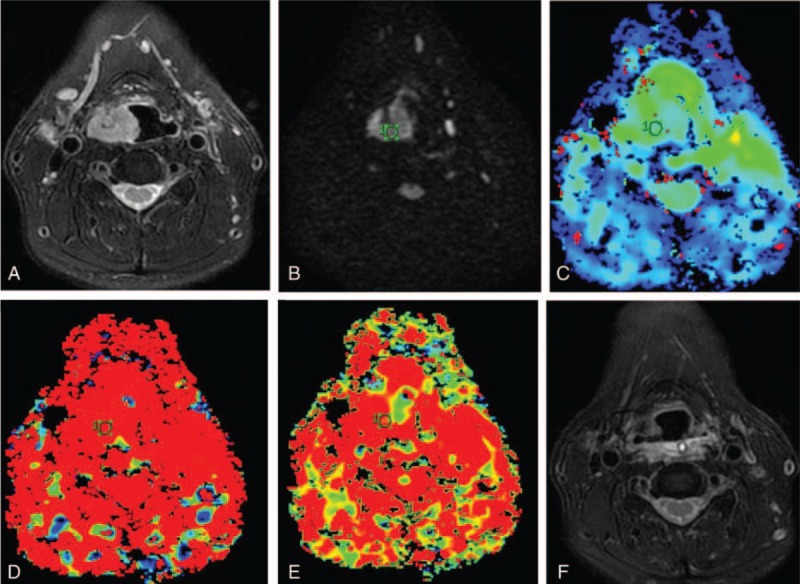
A 65-year-old man with right pyriform sinus carcinoma with good response to induction chemotherapy. (A) Transverse T_2_-weighted imaging. (B) Transverse diffusion weighted imaging (DWI) map obtained with *b* = 800 s/mm^2^. (C–E) The mean pretreatment *D*^∗^, *D*, and *f* values were 48.35 × 10^–3^ mm^2^/s, 0.775 × 10^–3^ mm^2^/s and 53.44% in responder, respectively. (F) Transverse T_2_-weighted imaging show that the mass disappeared completely after 2 cycle induction chemotherapy.DWI =  diffusion weighted imaging.

**FIGURE 2:**
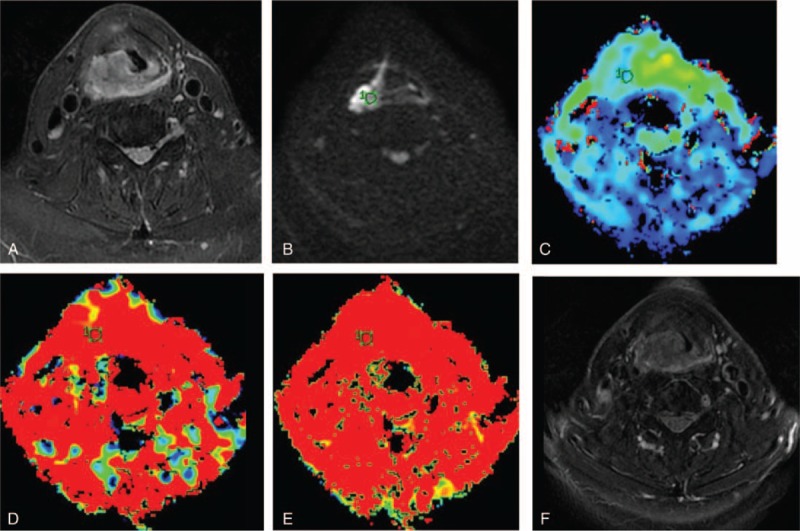
A 58-year-old man with pyriform sinus and posterior pharyngeal wall carcinoma, nonresponse to induction chemotherapy. (A) Transverse T_2_-weighted imaging. (B) Transverse diffusion weighted imaging (DWI) map obtained with *b* = 800 s/mm^2^. (C–E) The mean pretreatment *D*^∗^, *D*, and *f* values were calculated as 45.34 × 10^–3^ mm^2^/s, 0.872 ×10^–3^ mm^2^/s and 51.21% in nonresponder, respectively. (F) Transverse T_2_-weighted imaging show that tumor mass was slightly reduced (<30% reduction) after 2 cycle induction chemotherapy. DWI =  diffusion weighted imaging.

Based on the Response Evaluation Criteria in Solid Tumor criteria (RECIST), after 2 cycles of induction chemotherapy, 18 of 28 patients (64.3%) were categorized into the responder group (Figure 1), whereas the other 10 were considered nonresponders group (Figure 2). For primary tumors, the pretreatment ADC and *D* value in responders was significantly lower than in nonresponders (*P* = 0.018, 0.000; Table 1), whereas the pretreatment *D*^∗^ and *f* parameters between them had no significant changes (*P* = 0.242, 0.891; Table 1).

**TABLE 1:**
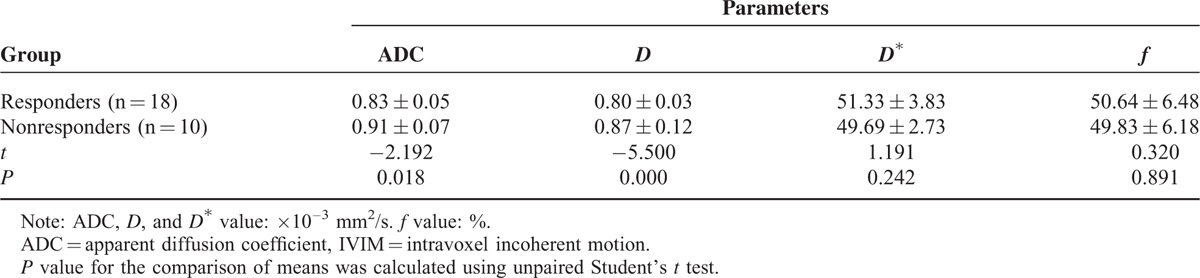
Comparison of Pretreatment IVIM Values and ADC Value Between Responders and Nonresponders

### Post-treatment IVIM-Derived Parameters in Different Response Groups

For primary tumors, the post-treatment ADC and *D* value in responders was significantly higher than in nonresponders (*P* =  0.024, 0.001; Table 2), the post-treatment *D*^∗^ value in responders was significantly lower than in nonresponders (*P* =  0.035; Table 2), whereas the post-treatment *f* parameters between them had no significant changes (*P* = 0.558; Table 2).

**TABLE 2:**
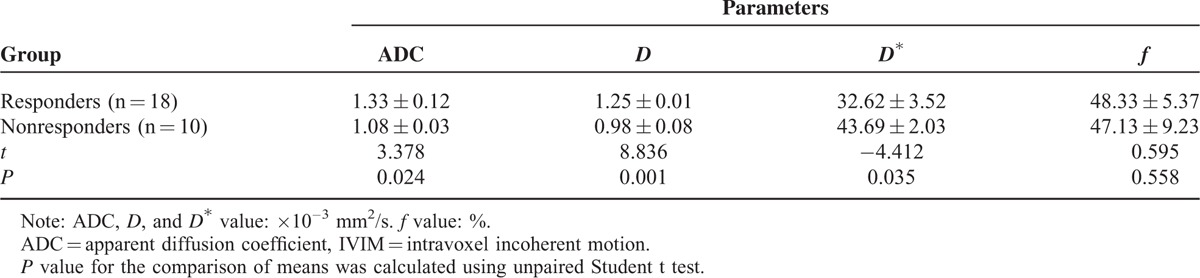
Comparison of Post-treatment IVIM Values and ADC Value Between Responders and Nonresponders

### Comparison of ADC and IVIM Values Between Before and After Treatment

For primary tumors, the ADC and *D* value in the post-treatment group was significantly higher than in the pretreatment group (*P* = 0.028, 0.006; Table 3), the *D*^∗^ in the post-treatment group was significantly lower than in the pretreatment group (*P* = 0.015; Table 3), whereas the *f* parameters between before and after treatment had no significant changes (*P* = 0.223; Table 3).

**TABLE 3:**
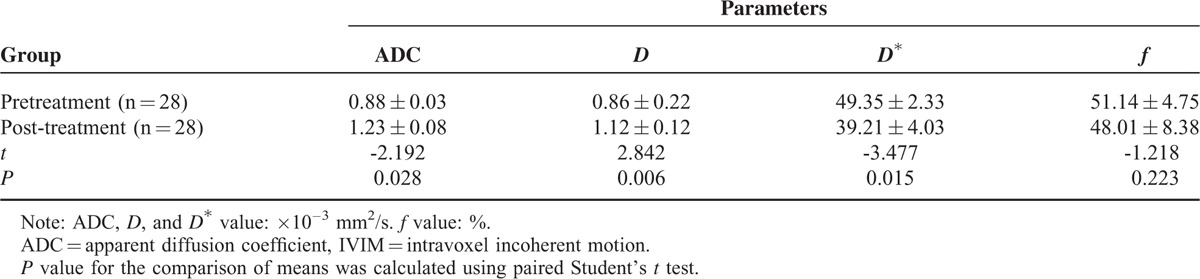
Comparison of IVIM Values and ADC Value Between Before and After Treatment

### Changes of ADC and IVIM Values Between Before and After Treatment in Different Response Groups

For primary tumors, the Δ ADC, Δ*D* and Δ*D*^∗^ in responders was significantly higher than in nonresponders (*P* = 0.026, 0.013, 0.002; Table 4), whereas the Δ *f* between them had no significant changes (*P* = 0.198; Table 4).

**TABLE 4:**
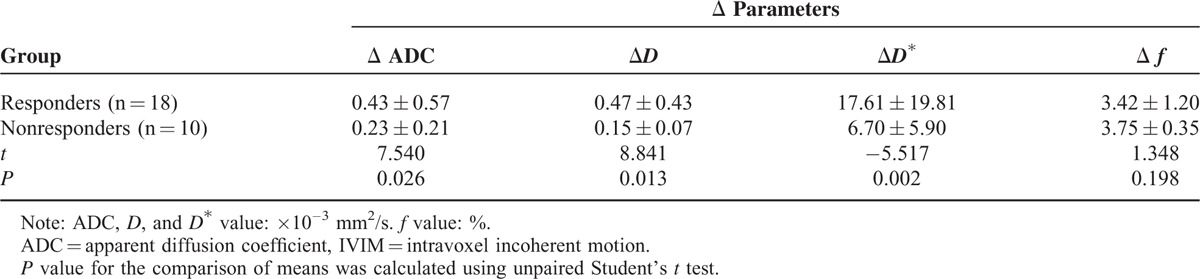
Changes of IVIM Values and ADC Value Between Before and After Treatment

### Comparison of Predictive Value of ADC and IVIM Values for Differentiation of Responders From Nonresponders

**FIGURE 3:**
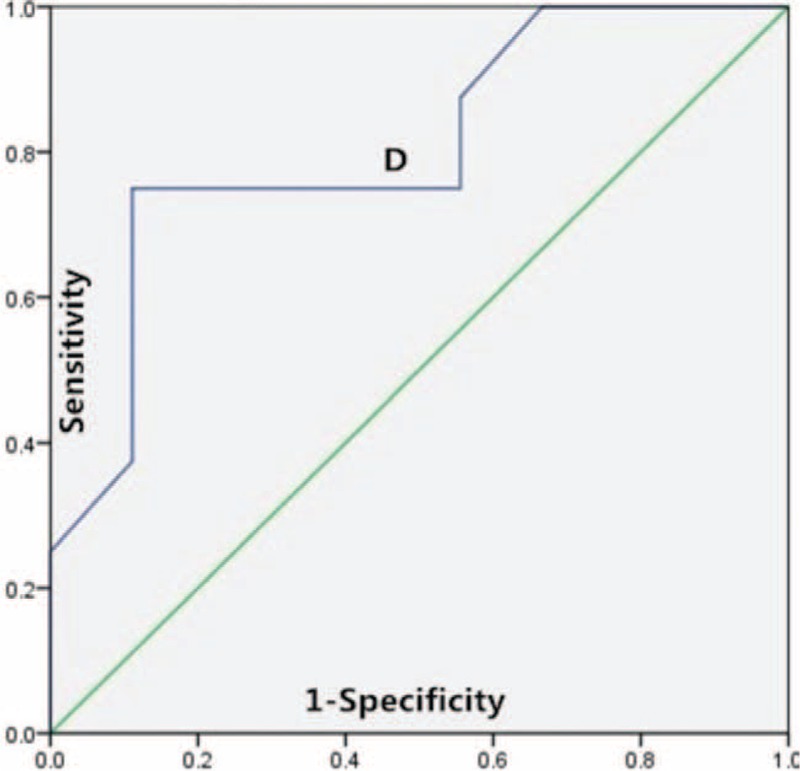
ROC curves for differentiation of responders from nonresponders based on the pretreatment *D* value. ROC = receiver operating characteristic.

The ROC curve analysis indicated that the areas under curve (AUC) of pretreatment ADC, *D* value, post-treatment ADC, *D*, and *D*^∗^ value, Δ ADC, Δ*D*, and Δ*D*^∗^ value were 0.778, 0.806, 0.775, 0.798, 0.655, 0.752, 0.678, and 0.796, respectively. The cutoff for pretreatment *D* value in best predicting responders was 0.847 × 10^−3^ mm^2^/s, and the corresponding AUC, sensitivity, and specificity were 0.806, 75.0%, and 88.9%, respectively (Figure 3).

### Pretreatment ADC and IVIM-Derived Parameters in Different Pathological Grade Groups

The pretreatment ADC and IVIM-derived parameters were as follows: G1: ADC = 0.83 ± 0.13 × 10^–3^ mm^2^/s, *D* = 0.83 ± 0.03 × 10^–3^ mm^2^/s, *D*^∗^ = 49.52 ± 4.10 × 10^–3^ mm^2^/s, *f* = 50.69 ± 4.44%.G2:ADC = 0.87 ± 0.07 × 10^–3^ mm^2^/s, *D* = 0.83 ± 0.04 × 10^–3^ mm^2^/s, *D*^∗^ = 50.83 ± 3.52 × 10^–3^ mm^2^/s, *f* = 50.86 ± 6.95%;G3:ADC =  = 0.88 ± 0.09 × 10^–3^ mm^2^/s, *D* = 0.82 ± 0.05 × 10^–3^ mm^2^/s, *D*^∗^ = 51.65 ± 3.04 × 10^–3^ mm^2^/s, *f* = 49.56 ± 7.29%.The pretreatment ADC, *D*, *D*^∗^, and *f* values had no significant differences between different pathological grading. Although *D*^∗^ value had no significant differences between G1, G2, and G3 group, a trend towards lower *D*^∗^ were observed with increasing tumor grading from G3 to G1 (Table 5).

**TABLE 5:**
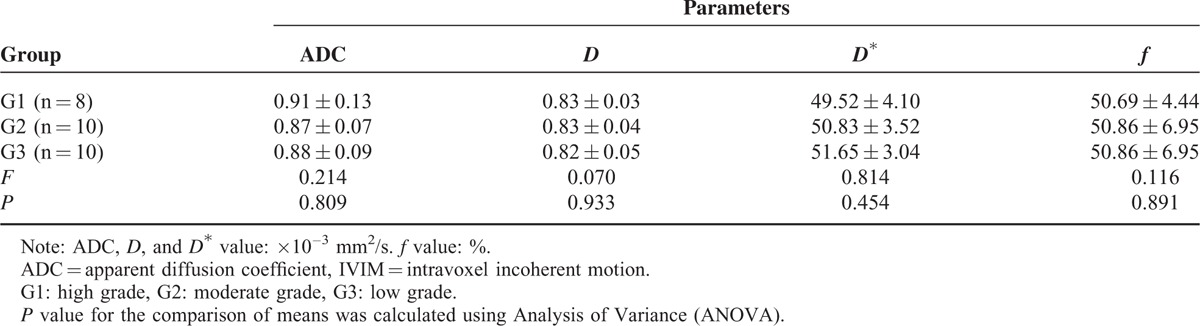
Comparison of Pretreatment IVIM Value and ADC Value Between Patients With Different Pathological Grading

## DISCUSSION

Our present study suggested that the post-treatment ADC value and *D* value was significantly higher than the pretreatment value and the post-treatment *D*^∗^ value was significantly lower than the pretreatment value (all *P* < 0.05); however no significant change in post-treatment *f* value was observed (*P* > 0.05). Compared with nonresponders, the lower pretreatment ADC, *D* value, and post-treatment *D*^∗^ value, and higher post-treatment ADC, *D* value, and higher ΔADC, Δ*D*, and Δ*D*^∗^ were observed in responders (all *P* < 0.05), but no significant change in Δ*f* was observed (*P* > 0.05). The ROC curve analysis indicated that pretreatment *D* value was the best parameter for predicting responders, when the cutoff value was 0.847 × 10^−3^ mm^2^/s, and the corresponding AUC, sensitivity, and specificity were 0.806, 75.0%, and 88.9%, respectively. By using the pretreatment *D* value can effectively differentiate the responders from nonresponders in locally advanced hypopharyngeal carcinoma.

In IVIM theory, molecular diffusion affected by the microcirculation of blood in the capillary network.^11^ Compared to the ADC value, the *D* value represents the true molecular diffusion and associates with the ratio of intracellular and extracellular spaces. After chemotherapy, with the tumor cell density and the restrictions of water molecules reduced, a higher ADC, a higher *D* value and changes of them will be observed. In contrast, diffusion is more restricted in highly cellular tumors may lead to a lower ADC and a lower *D* value. In the present study, patients with disease responsive to chemoradiation therapy had significantly lower pretreatment ADC, *D* value, higher post-treatment ADC, *D* value, ΔADC, and Δ*D* value from primary tumor than patients with no response. These results are consistent with the idea that tumor cell density in highly proliferating state in solid tumor may closely related to chemotherapy sensitivity. In the previous study, Hatakenaka et al^26^ reported that a lower ADC in primary cancer may predict good treatment response in patients with HNSCC underwent chemoradiation. Lu et al ^27^ reported that 16 patients with a lower *D* had prolonged progression-free survival (PFS) and overall survival (OS) in HNSCC. In the recent study, Lai et al^28^ and Xiao et al^29^ also demonstrated that a notably lower baseline *D* value and a higher changes of *D* value were observed in responders from chemotherapy in NPC tumors, which both agreed with our results. In addition, IVIM-derived parameters can also be confounded by tumor's heterogeneity, and tumor hypoxia in the early chemotherapy period, which can especially affect the measurement of ADC and *D* value, and contribute to an overlapping result.

The differentiation level of tumor was related to the prognosis and treatment options. The worse the differentiation of tumor was, the higher the malignant degree of tumor was, the tumor need more blood supply and the microcirculation perfusion of tumor will be increased, which is more sensitive to chemoradiation. The *D*^∗^ value can reflects the status of microcirculation perfusion of tumor, and it can provide quantitative evidence for clinical treatment. Our present study indicates that a lower post-treatment *D*^∗^ and a higher Δ*D*^∗^ may predict good response to induction chemotherapy in hypopharyngeal carcinoma. In the previous study, Zima et al^30^ reported that tumor with a high perfusion status might closely better to a good response in HNSCC, which agreed with our present results that the early changes of *D*^∗^ value can effectively distinguish responders from nonresponders, despite no significant differences in pretreatment *D*^∗^ value (*P* = 0.242). In addition, the pretreatment IVIM-derived parameters presented no significant differences between G1, G2, and G3 group, although a trend towards lower *D*^∗^ was observed with increasing tumor grading from G3 to G1 (51.65 ± 3.04 × 10^–3^ mm^2^/s vs 50.83 ± 3.52 × 10^–3^ mm^2^/s vs 49.52 ± 4.10 × 10^–3^ mm^2^/s, *P* = 0.454). Because of small sample size, there are some overlap in the pathological grading of G2 and G3 groups. The correlation between pretreatment *D*^∗^ value and the pathological grading of hypopharyngeal carcinoma was uncertain, which needs a further study.

The *f* value correlates with microvessel density (MVD) in normal or intact vasculature and reflects the speed of angiogenesis to some extent. Hauser et al^31,32^ suggested that a high initial *f* for primary cancer may predict poor treatment response to CRT in patients with HNSCC. On the contrary, by using DCE-MRI, Chawla et al ^33^ reported that tumor with a lower plasma volume fraction (PV) may predict a poor prognosis. In this present study, we found that the pretreatment *f* value and changes in *f* value have no significantly among the responders and nonresponders. Previous study reported that, under chemotherapy, the speed of angiogenesis grew more slowly and showed a lower density of newly formed blood vessels, normal angiogenesis should not change significantly at the early stage of induction chemotherapy,^34,35^ so an observation window of 42 days (2 cycle) may be enlarged. In addition, the tumor's heterogeneity, and the overlap between different pathological grading both can affect the measurement of *D*^∗^ and *f* values.

The first limitation of this study mainly evaluated the early response rather than the long-term prognosis. Second, we excluded 5 patients because of MR artifacts or poor visualization of the primary lesion. Although we take some effective measures that include codeine for oral administration before the MRI scanning and optimized scanning parameters (Dual Spin Echo technique was turned off before IVIM scanning) to reduce artifacts and increase the signal-to-noise ratio, the intrinsic limitations of IVIM in head and neck imaging were still challenging. In addition, the sample size of this study was relatively small. Finally, the choice and appropriate number of *b* values suitable for hypopharyngeal IVIM imaging are still unknown. So the selection of *b* value and numbers would then be an optimization procedure.

## CONCLUSIONS

The IVIM-derived parameters, especially pretreatment *D* value show promise as an imaging biomarker in predicting the induction chemotherapy response for locally advanced hypopharyngeal carcinoma.
